# Clinical Staging of Amyotrophic Lateral Sclerosis in Chinese Patients

**DOI:** 10.3389/fneur.2018.00442

**Published:** 2018-06-19

**Authors:** Xueping Chen, Qian-Qian Wei, Yongping Chen, Bei Cao, RuWei Ou, Yanbing Hou, Xiaoqin Yuan, Lingyu Zhang, Hui Liu, Huifang Shang

**Affiliations:** Department of Neurology, West China Hospital, Sichuan University, Chengdu, China

**Keywords:** amyotrophic lateral sclerosis, staging system, treatment, survival, riluzole

## Abstract

**Objective:** It is important to explore the utility of clinical staging systems in the management of amyotrophic lateral sclerosis (ALS). Our aim was to assess the validity of King's College in a Chinese ALS cohort, by evaluating the duration and informativeness of each stage and examining the association between stage and prognosis.

**Methods:** From May 2008 to December 2016, patients with a likely diagnosis of ALS were registered. We prospectively assessed the progression of the patients through the stages and calculated the duration of each stage.

**Results:** The median duration in Stage 1 was 12.00 months, Stage 2 7.50 months, Stage 3 6.50 months, and Stage 4 4.10 months. Subset analysis revealed that the spinal-onset and early-onset patients had a longer median time in Stage 1 compared to bulbar-onset and late-onset patients, respectively. Riluzole treatment extended the durations of Stages 1 and 2, and the effect was maintained in patients with long-term use of riluzole (>6 months). Patients who initiated long-term riluzole therapy early, in Stage 1 or 2, had a longer Stage 2. Patients who received percutaneous gastrostomy endoscopy (PEG) or non-invasive positive-pressure ventilation (NIPPV) showed longer durations of Stage 4. The differences in survival time measured from each stage to death or censor date were significant.

**Conclusions:** We validated the King's College staging system in a Chinese population, and showed this system to be useful in clinical practice. Patients with bulbar-onset or an age of onset>45 years tended to have rapidly progressing ALS. Riluzole may be more effective when initiated in an early disease stage and continued long-term. PEG and NIPPV treatments can extend disease duration of Stage 4.

## Introduction

Amyotrophic lateral sclerosis (ALS) is a fatal neurodegenerative disorder characterized by progressive degeneration of upper and lower motor neurons in the brain and spinal cord with a median survival of 3–5 years after diagnosis (1). The inter-individual variability of symptoms and progression is high in ALS, making prognosis and treatment challenging. Several prognostic factors have been increasingly investigated, including age of onset, onset form, diagnostic delay, ALS Functional Rating Scale-Revised (ALSFRS-R) score, and respiratory function. In addition, therapeutic interventions such as riluzole, percutaneous endoscopic gastrostomy (PEG), non-invasive positive-pressure ventilation (NIPPV), and tracheostomy have been found to be associated with prolonged survival (2–7). Recently, studies have reported that the presence of cognitive impairment and environmental effects may be related to survival in ALS (8–11).

None of the proposed ALS models includes milestones, however. Regression milestones in ALS are essential to establish accurate staging criteria, which in turn may help predict prognosis, provide a simple and objective measure of disease progression, and be useful for individualized counseling and drug effect evaluation (12). At present, there are 2 clinical staging systems for ALS, the “Milano-Torino” (MiToS) (13) and “King's College” (12) systems. The MiToS system uses six stages, from 0 to 5. It is based on the loss of independent function in four key domains assessed by the ALSFRS-R (14), with stage 0 being normal function and stage 5 being death. The King's College system uses five stages, from 1 to 5. It is based on disease burden and considers the number of involved regions for the first three stages and the significant feeding or respiratory failure when PEG and NIPPV are needed for the subsequent stages, with stage 1 being symptom onset and stage 5 being death. Studies have compared these two staging systems, and one study showed that the King's College staging system is able to differentiate early to mid-disease well with consideration of clinical or disease burden, and MiToS system is mostly skewed toward later disease stages with consideration of functional involvement. The MiToS system is based on the complete loss of function in different domains of the ALSFRS-R, and the King's College staging system is not relying on ALSFRS-R, but it can be estimated from the ALSFRS-R with 92% concordance (15). This staging system aimed more toward the distinction of functional capabilities during the spread of the disease, and it was shown to have higher homogeneity and discriminatory ability (16).

No studies have addressed staging of ALS patients in China; therefore we used the validated King's College staging system in a Chinese cohort to determine the length of each stage in Chinese ALS patients and the association between stage and prognosis. We also evaluated differences in stage duration among different ALS subgroups as well as in patients receiving different treatments.

## Methods

### Patients

This longitudinal and observational study was conducted at the tertiary referral center of South-West China (Department of Neurology, West China Hospital of Sichuan University). From May 2008 to December2016, patients in whom the neurologist suspected a likely diagnosis of ALS were asked to participate in the registry. Patients with a diagnosis of progressive muscular atrophy, progressive bulbar paralysis, or primary lateral sclerosis were excluded. At the moment of patient registration, demographical and clinical variables were collected. Early onset was defined as < 45 years of age. Onset forms were classified as spinal (upper or lower) or bulbar. Diagnostic delay was defined as the interval (in months) between the onset and diagnosis dates. Patients were considered as positive or negative to the use of riluzole based on the fact that they assumed riluzole 100 mg/day ≥ or < 1 month respectively. Those patients positive for the use of riluzole were further classified as long-term or short-term treatment depending on whether they assumed riluzole for ≥ or < 6 months respectively. Patients were followed up by telephone or in person at 3-month intervals by our neurologists. Information collected at follow-up reviews included disease progression, current clinical manifestations, medication, body weight, ALSFRS-R score, respiratory function, and clinical interventions. The timings of PEG and NIPPV treatment were recorded. Patients whom we were unable to contact for 2 successive follow-up reviews were assigned to the loss of follow-up group. All patients gave informed written consent prior to enrollment. This study was approved by the Ethics Committee of West China Hospital of Sichuan University.

### ALS staging

According to King's College staging system, ALS staging was based on the presence of wasting, weakness, spasticity, dysphagia, or dysarthria in different central nervous system regions defined as bulbar, upper limb, lower limb or diaphragmatic (12). Involvement of 1 CNS region defined as bulbar, upper or lower limb, or diaphragmatic was Stage 1. Functional involvement of a second region was Stage 2. Functional involvement of a third region was Stage 3. Swallowing impairment requiring gastrostomy and respiratory decline requiring non-invasive ventilation was Stage 4. Stage 5 was death. The King's College staging system defines Stage 2A as an ALS diagnosis and Stage 2B as functional involvement of a second region. However, a diagnosis may be made at virtually any point in the disease course, and Stages 2A and 2B may occur concurrently. Therefore, we merged Stages 2A and 2B into Stage 2, defined as functional involvement of a second region. Not all patients came to our center at early stages, and sometimes some patients had already shown significant involvement of respiratory muscles and NIPPV was required. For example, if a patient was already in Stage 3 when referred to our center, data on Stage 1 and Stage 2 durations were retrospectively collected. For patients who experienced each disease stage, the median duration of each stage was calculated. The median survival time was calculated separately for those who had died and for the whole population. For those who were still alive at the end of the study, they were considered censored for analysis of median survival time of the population.

### Statistical analysis

Comparisons of continuous variables between 2 groups were made using Student's *t*-test. One-way analysis of variance was used to compare variables for 3 or more groups. If the data were not parametrically distributed or their transformation did not result in normality, non-parametric tests were used to explore differences between groups. The χ^2^ test was used to compare categorical variables. Kaplan-Meier curves and log-rank tests were used, with survival measured from clinical stage to death or censor date. Analyses of covariance adjusted for confounding factors were performed to compare the variables in subgroups to avoid confounding interference. Subgroup analyses were conducted by sex, age of onset, onset form, use of riluzole, and acceptance of PEG or NIPPV. All variables were analyzed using descriptive statistics, including the median, mean, 95% confidence interval (CI), and inter-quartile range (IQR). All analyses were performed using the SPSS 18.0 statistical software. *P*-values of < 0.05 were considered to be statistically significant.

## Results

### Patient characteristics

A total of 1,696 patients were registered from May 2008 to December 2016. However, 41 patients with incomplete information or misdiagnosis were excluded, and another 182 patients failed to follow up. Therefore, a total of 1,473 patients with a completed clinical report form were included. At the end of the study, 864 patients had died and 609 patients were alive. The mean age at onset was 54.2 years (95%CI 53.6–54.8); 60.5% of the patients were male, 24.4% early-onset, and 21.8% bulbar-onset. The mean ALSFRS-R total score at baseline was 37.6 (95%CI 37.2–37.9, range 9–48). The diagnostic delay is 15.3 ± 14.1 months. The median survival time of all patients was 38.6 months (95%CI 36.6–40.6, range 5.3–94.5). For the deceased patients, the median survival time was 29.7 months (95%CI 28.6–30.8, range 5.3–68.7).

### Proportions of patients moving to each stage

The total number of patient who reached each milestone during the follow-up and the number of those who progressed from one milestone to another are displayed in Table 1. For patients who reached Stages 2, 3, 4, and 5, the majority progressed to the following stage; a small proportion (11.3–17.1%) skipped a stage as the disease progressed; 0.07–3.40% skipped multiple stages, therefore indicating a more aggressive form of the disease; only 689 patients experienced each disease stage (Stages 1–4). None moved backward to an earlier stage (Table 1). Patients with sequential progression tended to have a better prognosis than did stage-skipping patients (median survival time 33.5 months vs. 30.4 months, *p* = 0.011).

**Table 1:** Number and proportion of patients who reached each stage during the disease progression.

**Disease stage**	**Number**	**Number and % in Stage 1**	**Number and % in Stage 2**	**Number and % in Stage 3**	**Number and % in Stage 4**	**Number and % in Stage 5**	**Number and % remained**
**Stage 1**	1,473	–	1,145 (77.73%)	252 (17.11%)	27 (1.83%)	13 (0.07%)	36 (2.44%)
**Stage 2**	1,145	–	–	883 (77.12%)	129 (11.27%)	39 (3.40%)	94 (8.21%)
**Stage 3**	1,135	–	–	–	816 (71.89%)	145 (12.78%)	174 (15.33%)
**Stage 4**	972	–	–	–	–	667 (68.62%)	305 (31.38%)

### Duration of each stage and subset analysis

**Figure 1:**
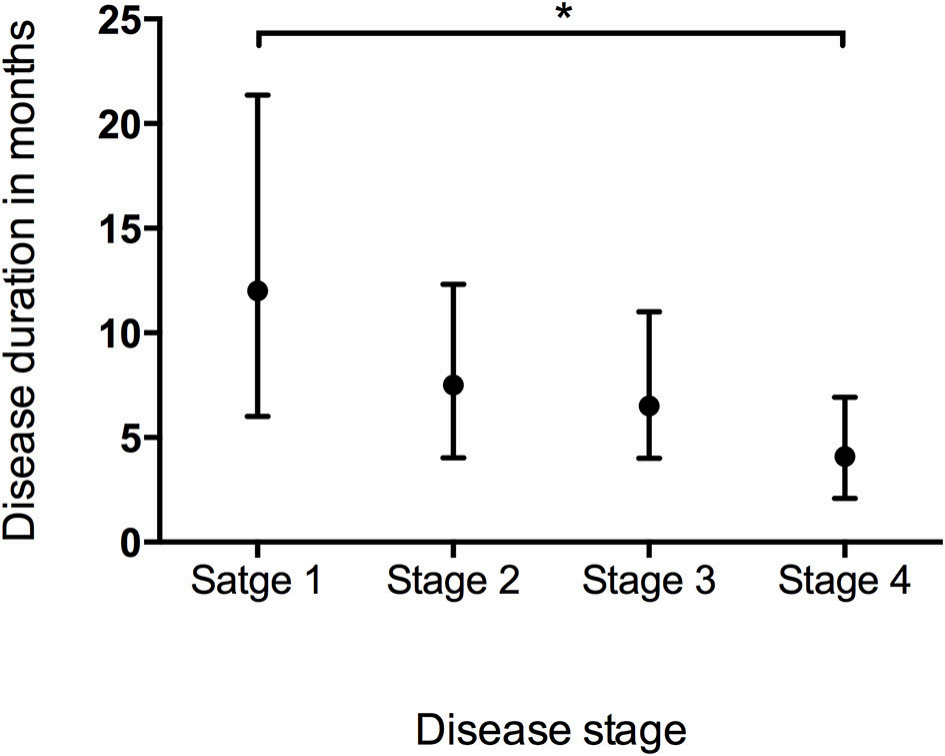
Box plot of duration in months spent at each stage representing median and range of values. ^*^P < 0.05.

**Figure 2:**
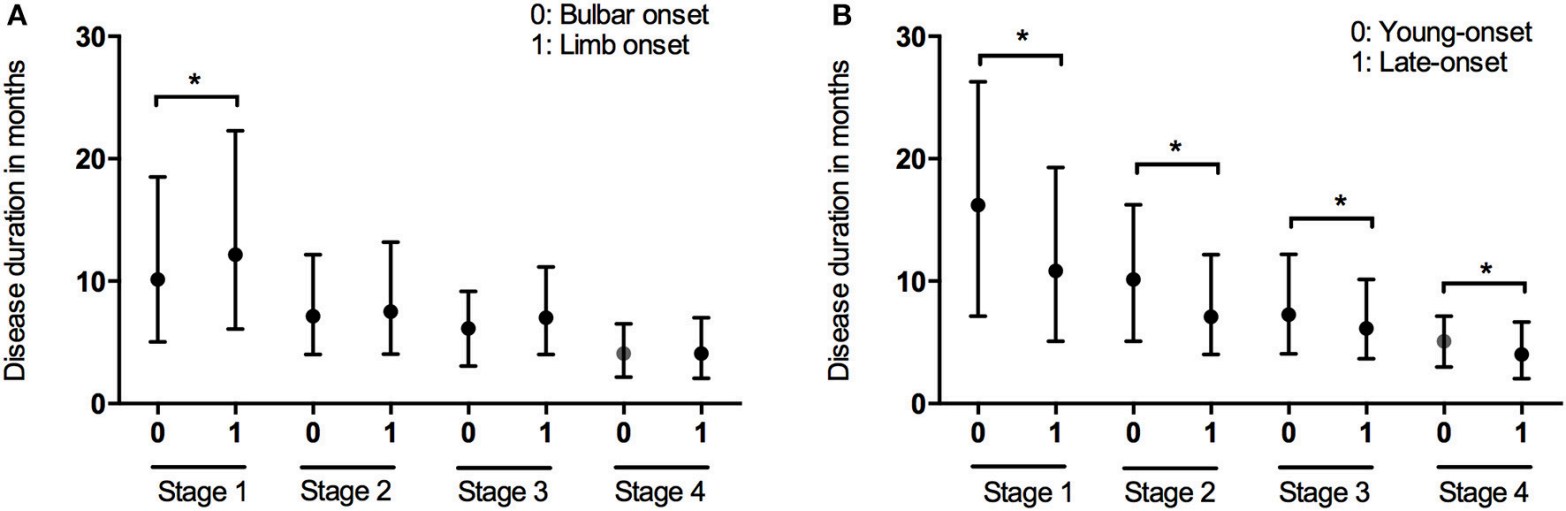
Box plot of duration in months spent at each stage in patients with different onset form **(A)** and onset of age **(B)**. ^*^P < 0.05.

Data of the patients who experienced each disease stage as ALS progressed were used to calculate the duration of every stage. The median time spent in any stage was 7.1 months (95%CI 6.7–7.1, IQR 4.0–12.2). The median times spent in a stage before progressing to the consecutive stage were as follows: Stage 1, 12.0 months (95%CI 10.8–12.2, IQR 6.0–21.4); Stage 2, 7.5 months (95%CI 7.1–8.1, IQR 4.0–12.3); Stage 3, 6.5 months (95%CI 6.1–7.1, IQR 4.0–11.0); Stage 4, 4.1 months (95%CI 4.0–5.0, IQR 2.1–6.9) (Figure 1). With the Bonferroni correction, the duration of Stage 1 was significantly longer than those of Stages 2, 3, and 4, and the duration of Stage 2 was significantly longer compared to those of Stages 3 and 4. Subgroup analysis revealed that the spinal-onset patients had a longer median time in Stage 1 than did the bulbar-onset patients (12.2 months vs. 10.1 months, *p* = 0.038) (Figure 2A and Table 2). The early-onset patients had a longer median time spent in each stage compared to the patients with an age of onset>45 years (Figure 2B and Table 2).

**Table 2:** Subgroups analysis in ALS patients.

	**Subgroup analysis**	**Median survival (months)**	**95% CI**	**IQR**	***P*-value**
Site of onset	Bulbar Spinal Bulbar Spinal Bulbar Spinal Bulbar Spinal	Stage 1 Stage 2 Stage 3 Stage 4	10.13 12.17 7.13 7.50 6.13 7.00 4.17 4.48	9.10–11.23 11.13–13.20 6.02–9.00 7.07–8.13 5.10–7.00 6.07–7.13 3.53–5.10 4.07–5.07	5.03–18.50 6.10–22.28 4.00–12.17 4.03–13.20 3.01–9.17 4.00–11.17 2.27–7.00 2.26–7.13	0.038^*^ 0.478 0.148 0.638
Onset age	Young-onset >45 years Young-onset >45 years Young-onset >45 years Young-onset >45 years	Stage 1 Stage 2 Stage 3 Stage 4	16.20 10.83 10.13 7.10 7.25 6.13 5.10 4.00	13.83–18.23 9.18–12.00 8.10–12.17 6.10–8.00 6.50–8.70 6.00–7.00 4.17–6.02 3.53–4.30	7.13–26.30 5.10–19.30 5.10–16.23 4.02–12.17 4.07–12.20 3.65–10.13 2.98–7.13 2.03–6.67	< 0.001^*^ < 0.001^*^ 0.002^*^ 0.020^*^
Riluzole	Treated Untreated Treated Untreated Treated Untreated Treated Untreated	Stage 1 Stage 2 Stage 3 Stage 4	13.10 11.13 8.13 7.13 7.07 6.13 4.10 4.57	11.13–15.25 10.13–12.17 7.13–10.35 6.13–8.10 6.03–8.10 6.03–7.08 4.00–5.00 4.07–5.10	6.10–24.22 5.10–19.30 4.08–14.52 4.00–12.17 3.98–12.17 4.00–10.10 2.23–7.13 2.30–7.06	0.026^*^ 0.014^*^ 0.158 0.924
Riluzole treatment	Long-term Short-term Long-term Short-term Long-term Short-term Long-term Short-term	Stage 1 Stage 2 Stage 3 Stage 4	15.47 11.05 10.57 7.07 7.13 6.80 4.50 4.07	12.20–20.00 7.13–13.20 8.00–12.17 6.05–9.13 5.10–9.55 5.10–8.10 3.53–6.00 3.07–5.07	8.10–26.30 5.10–19.30 5.10–16.26 4.00–12.20 4.00–12.60 3.73–11.13 2.27–7.13 2.10–7.13	0.009^*^ 0.007^*^ 0.265 0.890
Long-term treatment	Early stage Late Stage Early stage Late Stage Early stage Late Stage Early stage Late Stage	Stage 1 Stage 2 Stage 3 Stage 4	19.20 12.20 11.93 7.13 8.17 5.07 4.75 3.47	13.20–22.27 9.18–19.30 8.13–15.13 5.10–12.00 6.07–11.00 5.00–9.08 3.82–6.10 2.53–6.05	8.13–25.87 6.07–26.67 5.55–18.32 4.00–13.23 4.07–13.68 3.00–11.17 2.47–7.28 2.05–7.35	0.227 0.019^*^ 0.107 0.657
Short-term treatment	Early stage Late Stage Early stage Late Stage Early stage Late Stage Early stage Late Stage	Stage 1 Stage 2 Stage 3 Stage 4	10.10 12.65 8.50 7.06 6.30 7.00 4.07 4.00	7.13–13.10 6.00–17.27 6.57–11.13 5.96–8.17 4.10–8.17 5.00–9.17 3.03–5.57 3.00–5.67	6.08–18.50 4.00–21.02 5.09–12.20 4.00–12.00 3.25–11.12 4.00–11.18 2.55–7.48 2.03–7.13	0.699 0.304 0.880 0.740
PEG	Yes No	Stage 4	13.20 4.07	9.10–16.20 4.00–4.50	8.30–16.30 2.07–6.60	< 0.001^*^
NIPPV	Yes No	Stage 4	8.30 4.10	6.70–11.05 4.00–4.57	6.00–14.18 2.10–6.72	< 0.001^*^

ALS, amyotrophic lateral sclerosis; CI, confidence interval; IQR, inter-quartile range; PEG, gastrostomy percutaneous endoscopy; NIPPV, non-invasive positive pressure ventilation.

* P < 0.05.

### Treatments

**Figure 3:**
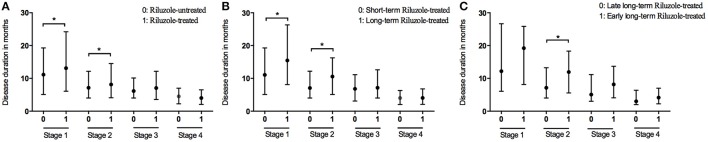
Box plot of duration in months spent at each stage between patients with treatment of riluzole and patient without treatment of riluzole **(A)**; between patients with long-term treatment of riluzole and patient with short-term treatment of riluzole **(B)**; between patients with early long-term treatment of riluzole and patient with late long-term treatment of riluzole **(C)**. ^*^P < 0.05.

At the end of the follow-up period, 521 patients (35.4%) were on riluzole. We analyzed the disease durations from patients who reached Stage 5, therefore among these patients, 474 patients didn't receive riluzole treatment, 122 patients used riluzole 100 mg/day for 1–6 months (short-term), and 124 patients used riluzole 100 mg/day for ≥6 months (long-term). The durations of Stages 1 and 2 were significantly longer in the riluzole-treated patients than those in the untreated patients (Figure 3A and Table 2), and the clinical features including diagnostic delay (15.6 ± 11.8 vs. 14.3 ± 13.3, *p* = 0.170), and age (55.4 ± 11.4 vs. 55.1 ± 11.4, *p* = 0.672), and site of onset (spinal/bulbar 193/53 vs. 474/144, *p* = 0.653) were similar between these two groups. To evaluate the effect of drug administration time, we compared the duration of each stage between 124 patients with long-term riluzole treatment and 122 patients with short-term treatment, who had similar disease characteristics including diagnostic delay (16.8 ± 11.6 vs. 14.2 ± 10.5, *p* = 0.062), and age (54.4 ± 11.2 vs. 54.9 ± 11.3, *p* = 0.716) and site of onset (spinal/bulbar 95/29 vs. 98/24, *p* = 0.536). The patients with long-term riluzole use had significantly longer durations of Stages 1 and 2 than did the short-term riluzole patients (Figure 3B and Table 2). Therefore, the survival was significantly longer in the long-term treatment group compared to that in the short-term treatment group (41.2 months vs. 33.0 months, *p* = 0.012). Furthermore, 81 patients started long-term treatment in Stage 1 or 2, and 43 patients in Stage 3 or 4. Patients who initiated long-term treatment early, in Stage 1 or 2, had a significantly longer duration of Stage 2 (11.9 months vs. 7.1 months, *p* = 0.019) (Figure 3C) and longer survival (45.3 months vs. 35.1 months, *p* = 0.035) compared to those of the patients who started long-term treatment late, in Stage 3 or 4.

**Figure 4:**
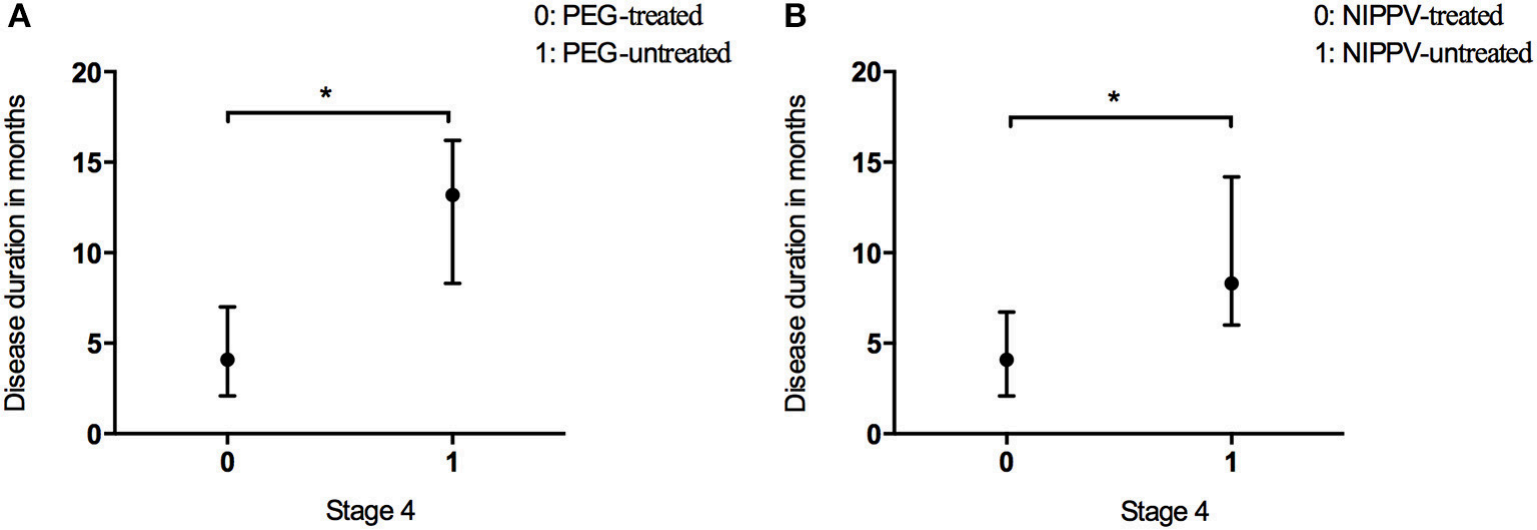
Box plot of duration in months spent at stage 4 between patients who received PEG **(A)** or NIPPV **(B)** treatment and patients who did not. ^*^P < 0.05.

In the present study, 2.9% of patients underwent PEG treatment, and 7.9% NIPPV treatment. The time spent in Stage 4 was longer in the patients undergoing PEG than that in the patients who did not receive PEG treatment (Figure 4A and Table 2). The time spent in Stage 4 was longer in the patients treated with NIPPV than that in the patients without NIPPV treatment (Figure 4B and Table 2).

### Validity of the staging system

**Figure 5:**
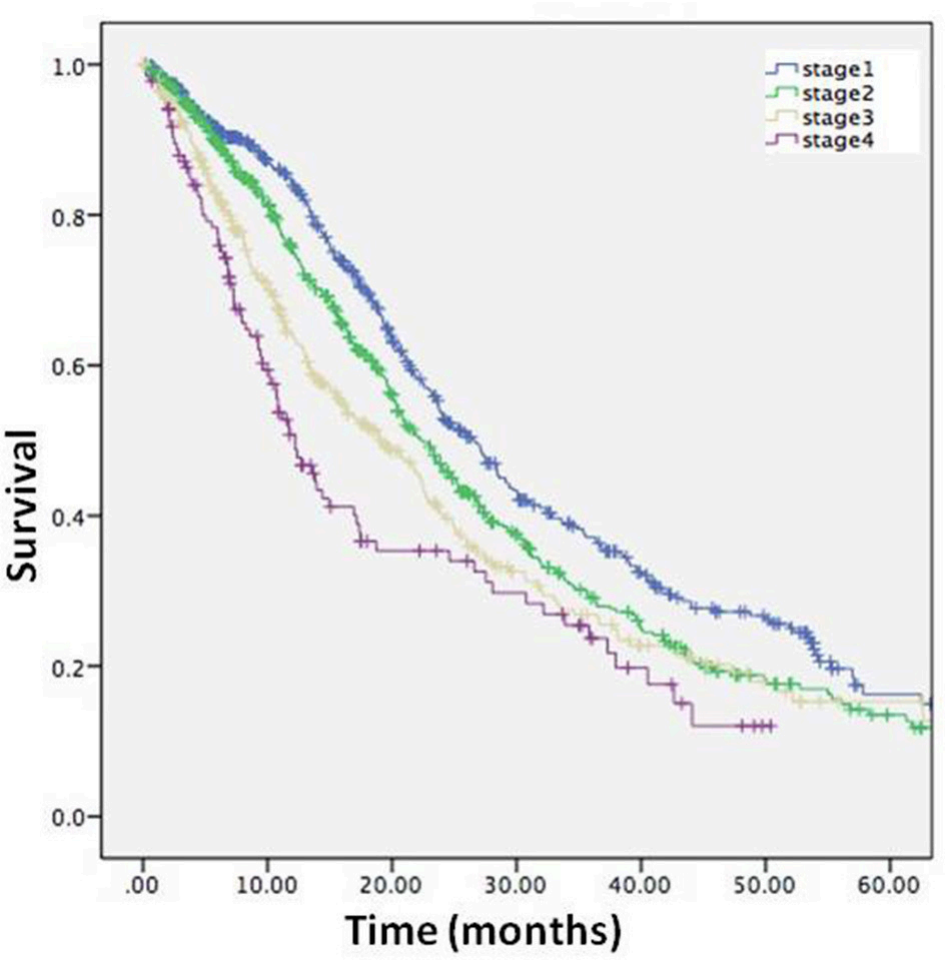
Kaplan-Meier curve describing survival for entire cohort from each stage to death or censor.

The survival time from the clinical stage to death or censor date was calculated in the entire cohort. The survival time were as follows: Stage 1, 22.6 months (95%CI 21.2–24.1); Stage 2, 20.1 months (95%CI 18.7–21.5); Stage 3, 17.1 months (95%CI 15.4–18.7); Stage 4, 13.7 months (95%CI 11.5–15.9), and the results of Kaplan-Meier survival analysis showed that survival time was affected by stage (log-rank *p* < 0.001) (Figure 5), and confirmed the validity of the staging system.

## Discussion

The findings of the present study have further validated the utility of the King's College staging system for patients with ALS in a Chinese ALS cohort, establishing that this staging system can be successfully used in clinical practice and trials. The King's College staging system is easy to apply and therefore can be used to quickly evaluate the health status of a patient with ALS (17). Furthermore, once an ALS patient is assigned to a certain stage, different types of institutional and professional care will be provided to the patient, helping allocate resources appropriately.

The King's College clinical staging system can potentially be utilized to formulate secondary endpoints in future clinical trials. Usually, death is used as an endpoint in ALS clinical trials (18–20). However, trials with survival as the primary outcome measures are typically 18 months in duration, since a minimum of 18 months of follow-up is required to obtain sufficient statistical power to reveal effects on a survival endpoint (21). The use of stage duration as a secondary endpoint can shorten and reduce the cost of a clinical trial. In addition, using death as an endpoint cannot answer the question which stage(s) is affected. In the present study, we found that Stage 1 had the longest duration in ALS patients. Therefore, Stage 1 allows a long therapeutic time window for a drug to be effective. If a drug prolongs Stage 1 or 2, it can be considered a successful intervention, since the quality of life in the earlier stages is much better than that in the later stages. In the ALS cohort overall, the subgroups with different clinical stage could be distinguished, and the survival time was significantly different. The separation of the curves is evidence of validity of the constructed system for staging.

ALSFRS-R score is commonly used as a secondary endpoint in clinical trials. While ALSFRS-R tracks progression of patients' disability (14, 22), King's College is meant to evaluate the anatomical extension of the disease. As a consequence, King's College could be particular useful in milder stages when weakness is still not causing any disability. On the contrary, MiTos system has been demonstrated to have a higher resolution in later stages of the disease (23). Despite ALSFRS-R is much more diffuse in clinical practice, both King's College and MiTos systems could be assessed basing on ALSFRS-R items scores (13, 17). Therefore, both King's College and MiTos systems could be used in complementarity in clinical practice, allowing a concise summary of the anatomical and functional burden of the disease.

The present study confirmed that each ALS stage was reached only after symptoms of weakness in a certain region had occurred, and no reversion to earlier stages was observed according to the King's College staging system. This finding is consistent with the clinical characteristics of ALS, since it begins insidiously with focal weakness but spreads quickly and irreversibly to involve most muscles. Furthermore, we found that median survival time was longer in patients with sequential progression than that in stage-skipping patients. Therefore, distinguishing stage-skipping phenotypes from the typical sequentially progressing ALS phenotype could have implications for clinical trials of putative disease-modifying therapies.

This longitudinal and observational study estimated the transition times between the clinical stages of ALS more accurately compared to the previous retrospective studies. In one such study, analyzing data from 2 large phase III clinical trials, it was difficult to measure the exact duration of Stage 1 because the data used to estimate the transition time from Stage 1 to Stage 2 were only collected after enrollment, which occurred after diagnosis (15). The authors found that the median time spent in Stage 1 was 18.1 months (15), which was longer than the median duration of Stage 1 in our study (12.00 months). In clinical trials the diagnosis of ALS trends to occur at the same time as Stage 2, but before diagnosis the patients already had shown symptoms of two regions. Therefore, the Stage 1 duration was artefacually long in the retrospective study (15). Deterioration has been shown to be non-linear in ALS, with the most rapid rates of decline occurring in the earliest and latest phases of the illness (24). In the present study, we did not find rapid progression in Stage 1; however, we did observe the most rapid progression in Stage 4, with the shortest median duration of 4.10 months. A possible explanation is that the rate of decline was calculated by ALSFRS-R, which has been shown to decline in a curvilinear way.

We also analyzed the effect of riluzole on the duration of each stage. Use of riluzole and long-, but not short-term riluzole treatment had positive effects on the time spent in Stages 1 and 2. Furthermore, we found that ALS patients who initiated long-term riluzole therapy in Stage 1 or 2 had a significantly longer Stage 2 and survival time compared to patients who started long-term treatment in Stage 3 or 4. These findings show that long-term (≥6 months) riluzole treatment is more effective than short-term (< 6months) treatment, and that riluzole should be started as early in the course of the disease as possible (Stage 1 or 2). One study in China also found that the use of riluzole for ≥6 months might have a positive effect on survival in ALS (25). In addition, several studies have reported the effect of riluzole to be more prominent in ALS patients in the early disease stages, and less pronouncedin patients in severe or advanced states (26–30). Although riluzole is indicated to slow the course of ALS in any stage (27, 31, 32), our study showed that riluzole was the most efficient in the early stages, significantly extending the times spent in Stages 1 and 2, when the disability is relatively mild.

In the subgroup analysis, we found that the spinal-onset patients had a longer median duration in Stage 1 compared to the bulbar-onset patients. A previous study showed that the time from the symptom onset to PEG was shorter in the bulbar-onset group than that in the spinal-onset group (33). Similarly, another study demonstrated that the time from the onset to NIPPV was shorter in the bulbar-onset group compared to the spinal- and flail-limb groups (34). This result indicates that ALS patients with bulbar onset may progress faster in the early stages of ALS. These patients were more strongly impaired by dysphagia than those with spinal-onset as disease progressed, and thus received intervention with gastrostomy sooner. Stage 4 duration was similar between the patients with spinal- onset and those with bulbar-onset in the present study; however, another study showed that the time from Stage 4A to Stage 5 was longer in bulbar-onset patients than that in spinal-onset patients (15). This discrepancy may be partly caused by the small number of patients with bulbar onset (43) who received PEG treatment in our cohort. However, we found that the time from Stage 4 to death was significantly longer in patients who had undergone PEG treatment compared to those who had not. Similarly, patients who had received NIPPV treatment showed a longer time from Stage 4 to death than did patients without NIPPV. A previous study showed that the median survival time from the symptom onset was significantly longer in NIPPV-treated patients than that in untreated patients (34); however, another study reported the opposite result (7). Our previous study using a Kaplan-Meier analysis did not find NIPPV to affect survival time, albeit an adjusted Cox proportional hazard model showed NIPPV to have a positive effect on the survival rate (35). Similarly, another study identified NIPPV as a significant survival-promoting factor with Cox proportional hazard regression after adjusting for various cofactors (36). A study in Taiwan using multivariate logistic regression also identified NIPPV as a predictor of long-term survival (4). In this study, NIPPV affected Stage 4 duration the most. The American Academy of Neurology 1999 (37) and 2009 (38) guidelines, and the European Federation of Neurological Societies (EFNS) 2005 (39) and 2012 (40) guidelines recommend the use of NIPPV and PEG to maintain quality of life, and to extend the life of patients with ALS. Nonetheless, no clear criteria exist for the timing of NIPPV in patients with ALS (40). Thus, the impact of earlier NIPPV treatment on survival is not clear, and further studies are required.

Several limitations of the present study should be noted. First, our results may have been affected by a potential sample selection bias since cases were ascertained solely through a single tertiary referral center. In addition, we excluded familial patients and other ALS populations. Patients were recruited when they were suspected to have ALS rather than according to the E1 Escorial diagnostic criteria, allowing for the possibility of misdiagnosis. Nonetheless, ALS mimics were excluded during the follow-up. Second, other factors may also be involved in disease progression, such as cognitive status, neuropsychological features, and pulmonary function, none of which was investigated at the time of this study. Third, even though an association between genetic variants and different clinical ALS profiles has been identified in several studies (1, 36), we did not explore the influence of genetic variants on the staging system.

In conclusion, the present study validates the King's College staging system in a Chinese population, and shows it to be useful in clinical practice and trials. ALS patients with bulbar-onset progressed faster in the middle stages of ALS compared to patients with spinal-onset. ALS patients with PEG or NIPPV treatments had a longer duration of Stage 4 compared to patients who didn't received these treatments. ALS patients who start long-term use of riluzole early in the course of the disease had a longer Stage 2 and survival time compared to those who started long-term treatment in late stage.

## Author contributions

XC, Q-QW, YC, BC, RO, and HS were involved in the conception, design, and data interpretation. All authors were involved in the collection of data. XC and Q-QW were involved in the drafting of the paper. All authors approved the final version.

### Conflict of interest statement

The authors declare that the research was conducted in the absence of any commercial or financial relationships that could be construed as a potential conflict of interest.

## References

[1] Brown RH Jr Al-Chalabi A Amyotrophic lateral sclerosis. N Engl J Med. (2017) 377:1602 10.1056/NEJMc1710379 29045202

[2] Bourke SC Tomlinson M Williams TL Bullock RE Shaw PJ Gibson GJ. Effects of non-invasive ventilation on survival and quality of life in patients with amyotrophic lateral sclerosis: a randomised controlled trial. Lancet Neurol. (2006) 5:140–7. 10.1016/S1474-4422(05)70326-4 16426990

[3] Chio A Calvo A Ghiglione P Mazzini L Mutani R Mora G. Tracheostomy in amyotrophic lateral sclerosis: a 10-year population-based study in Italy. J Neurol Neurosurg Psychiatry (2010) 81:1141–3. 10.1136/jnnp.2009.175984 20660920

[4] Lee CT Chiu YW Wang KC Hwang CS Lin KH Lee IT. Riluzole and prognostic factors in amyotrophic lateral sclerosis long-term and short-term survival: a population-based study of 1149 cases in Taiwan. J Epidemiol. (2013) 23:35–40. 10.2188/jea.JE20120119 23117224 PMC3700231

[5] Rooney J Byrne S Heverin M Corr B Elamin M Staines A. Survival analysis of irish amyotrophic lateral sclerosis patients diagnosed from 1995-2010. PLoS ONE (2013) 8:e74733. 10.1371/journal.pone.0074733 24098664 PMC3786977

[6] Stevic Z Kostic-Dedic S Peric S Dedic V Basta I Rakocevic-Stojanovic V. Prognostic factors and survival of ALS patients from Belgrade, Serbia. Amyotroph Lateral Scler Frontotemporal Degener. (2016) 17:508–14. 10.1080/21678421.2016.1195410 27315438

[7] Calvo A Moglia C Lunetta C Marinou K Ticozzi N Ferrante GD. Factors predicting survival in ALS: a multicenter Italian study. J Neurol. (2017) 264:54–63. 10.1007/s00415-016-8313-y 27778156

[8] Mitchell JD. Amyotrophic lateral sclerosis: toxins and environment. Amyotroph Lateral Scler Other Motor Neuron Disord. (2000) 1:235–50. 10.1080/14660820050515061 11465017

[9] Das K Nag C Ghosh M. Familial, environmental, and occupational risk factors in development of amyotrophic lateral sclerosis. N Am J Med Sci. (2012) 4:350–5. 10.4103/1947-2714.99517 22912943 PMC3421913

[10] Vinceti M Fiore M Signorelli C Odone A Tesauro M Consonni M. Environmental risk factors for amyotrophic lateral sclerosis: methodological issues in epidemiologic studies. Ann Ig. (2012) 24:407–415. 23193897

[11] Keren N Scott KM Tsuda M Barnwell J Knibb JA Ellis CM. Evidence of an environmental effect on survival in ALS. Amyotroph Lateral Scler Frontotemporal Degener. (2014) 15:528–33. 10.3109/21678421.2014.911326 24862874

[12] Roche JC Rojas-Garcia R Scott KM Scotton W Ellis CE Burman R. A proposed staging system for amyotrophic lateral sclerosis. Brain (2012) 135:847–52. 10.1093/brain/awr351 22271664 PMC3286327

[13] Chio A Hammond ER Mora G Bonito V Filippini G. Development and evaluation of a clinical staging system for amyotrophic lateral sclerosis. J Neurol Neurosurg Psychiatry (2015) 86:38–44. 10.1136/jnnp-2013-306589 24336810

[14] Cedarbaum JM Stambler N Malta E Fuller C Hilt D Thurmond B. The ALSFRS-R: a revised ALS functional rating scale that incorporates assessments of respiratory function. BDNF ALS Study Group (Phase III). J Neurol Sci (1999) 169:13–21. 10.1016/S0022-510X(99)00210-5 10540002

[15] Balendra R Jones A Jivraj N Steen IN Young CA Shaw PJ. Use of clinical staging in amyotrophic lateral sclerosis for phase 3 clinical trials. J Neurol Neurosurg Psychiatry (2015) 86:45–9. 10.1136/jnnp-2013-306865 24463480

[16] Ferraro D Consonni D Fini N Fasano A Del Giovane C Mandrioli J. Amyotrophic lateral sclerosis: a comparison of two staging systems in a population-based study. Eur J Neurol. (2016) 23:1426–32. 10.1111/ene.13053 27238551

[17] Balendra R Jones A Jivraj N Knights C Ellis CM Burman R. Estimating clinical stage of amyotrophic lateral sclerosis from the ALS Functional Rating Scale. Amyotroph Lateral Scler Frontotemporal Degener. (2014) 15:279–84. 10.3109/21678421.2014.897357 24720420

[18] Mitsumoto H Gordon P Kaufmann P Gooch CL Przedborski S Rowland LP. Randomized control trials in ALS: lessons learned. Amyotroph Lateral Scler Other Motor Neuron Disord. (2004) 5(Suppl. 1):8–13. 10.1080/17434470410019942 15512861

[19] Meininger V. Clinical trials in ALS: what did we learn from recent trials in humans? Neurodegener Dis. (2005) 2:208–14. 10.1159/000089627 16909027

[20] Berry JD Cudkowicz ME. New considerations in the design of clinical trials for amyotrophic lateral sclerosis. Clin Investig (2011) 1:1375–89. 10.4155/cli.11.127 22545191 PMC3335743

[21] Lanka V Cudkowicz M. Therapy development for ALS: lessons learned and path forward. Amyotroph Lateral Scler. (2008) 9:131–40. 10.1080/17482960802112819 18574756

[22] Proudfoot M Jones A Talbot K Al-Chalabi A Turner MR. The ALSFRS as an outcome measure in therapeutic trials and its relationship to symptom onset. Amyotroph Lateral Scler Frontotemporal Degener. (2016) 17:414–25. 10.3109/21678421.2016.1140786 26864085 PMC4950444

[23] Fang T Al Khleifat A Stahl DR Lazo La Torre C Murphy C Young C. Comparison of the King's and MiToS staging systems for ALS. Amyotroph Lateral Scler Frontotemporal Degener. (2017) 18:227–32. 10.1080/21678421.2016.1265565 28054828 PMC5425622

[24] Gordon PH Cheng B Salachas F Pradat PF Bruneteau G Corcia P Progression in ALS is not linear but is curvilinear. J Neurol. (2010) 257:1713–7. 10.1007/s00415-010-5609-1 20532545

[25] Chen L Liu X Tang L Zhang N Fan D. Long-term use of riluzole could improve the prognosis of sporadic amyotrophic lateral sclerosis patients: a real-world cohort study in China. Front Aging Neurosci. (2016) 8:246. 10.3389/fnagi.2016.00246 27822184 PMC5075535

[26] Riviere M Meininger V Zeisser P Munsat T. An analysis of extended survival in patients with amyotrophic lateral sclerosis treated with riluzole. Arch Neurol. (1998) 55:526–8. 10.1001/archneur.55.4.526 9561981

[27] Bensimon G Lacomblez L Delumeau JC Bejuit R Truffinet P Meininger V A study of riluzole in the treatment of advanced stage or elderly patients with amyotrophic lateral sclerosis. J Neurol. (2002) 249:609–15. 10.1007/s004150200071 12021952

[28] Traynor BJ Alexander M Corr B Frost E Hardiman O. An outcome study of riluzole in Amyotrophic Lateral Sclerosis–a population-based study in Ireland, 1996-2000. J Neurol. (2003) 250:473–9. 10.1007/s00415-003-1026-z 12700914

[29] Zoing MC Burke D Pamphlett R Kiernan MC. Riluzole therapy for motor neurone disease: an early Australian experience (1996-2002). J Clin Neurosci. (2006) 13:78–83. 10.1016/j.jocn.2004.04.011 16410201

[30] Zoccolella S Beghi E Palagano G Fraddosio A Guerra V Lepore V. ALS multidisciplinary clinic and survival. Results from a population-based study in Southern Italy. J Neurol. (2007) 254:1107–12. 10.1007/s00415-006-0401-y 17431705

[31] Bensimon G Lacomblez L Meininger V. A controlled trial of riluzole in amyotrophic lateral sclerosis. ALS/Riluzole Study Group. N Engl J Med. (1994) 330:585–91. 10.1056/NEJM199403033300901 8302340

[32] Paillisse C Lacomblez L Dib M Bensimon G Garcia-Acosta S Meininger V. Prognostic factors for survival in amyotrophic lateral sclerosis patients treated with riluzole. Amyotroph Lateral Scler Other Motor Neuron Disord. (2005) 6:37–44. 10.1080/14660820510027035 16036424

[33] Bokuda K Shimizu T Imamura K Kawata A Watabe K Hayashi M. Predictive factors for prognosis following unsedated percutaneous endoscopic gastrostomy in ALS patients. Muscle Nerve (2016) 54:277–83. 10.1002/mus.25051 26799526

[34] Berlowitz DJ Howard ME Fiore J.F. Jr. Vander Hoorn S O'donoghue FJ Westlake J. Identifying who will benefit from non-invasive ventilation in amyotrophic lateral sclerosis/motor neurone disease in a clinical cohort. J Neurol Neurosurg Psychiatry (2016) 87:280–6. 10.1136/jnnp-2014-310055 25857659

[35] Wei Q Chen X Zheng Z Guo X Huang R Cao B. The predictors of survival in Chinese amyotrophic lateral sclerosis patients. Amyotroph Lateral Scler Frontotemporal Degener. (2015) 16:237–44. 10.3109/21678421.2014.993650 25581512

[36] Burkhardt C Neuwirth C Sommacal A Andersen PM Weber M. Is survival improved by the use of NIV and PEG in amyotrophic lateral sclerosis (ALS)? A post-mortem study of 80 ALS patients. PLoS ONE (2017) 12:e0177555. 10.1371/journal.pone.0177555 28542233 PMC5441602

[37] Miller RG Rosenberg JA Gelinas DF Mitsumoto H Newman D Sufit R. Practice parameter: the care of the patient with amyotrophic lateral sclerosis (an evidence-based review): report of the Quality Standards Subcommittee of the American Academy of Neurology: ALS practice parameters task force. Neurology (1999) 52:1311–23. 10.1212/WNL.52.7.1311 10227612

[38] Miller RG Jackson CE Kasarskis EJ England JD Forshew D Johnston W. Practice parameter update: the care of the patient with amyotrophic lateral sclerosis: multidisciplinary care, symptom management, and cognitive/behavioral impairment (an evidence-based review): report of the Quality Standards Subcommittee of the American Academy of Neurology. Neurology (2009) 73:1227–33. 10.1212/WNL.0b013e3181bc01a4 19822873 PMC2764728

[39] Andersen PM Borasio GD Dengler R Hardiman O Kollewe K Leigh PN. EFNS task force on management of amyotrophic lateral sclerosis: guidelines for diagnosing and clinical care of patients and relatives. Eur J Neurol. (2005) 12:921–38. 10.1111/j.1468-1331.2005.01351.x 16324086

[40] Andersen PM Abrahams S Borasio GD De Carvalho M Chio A Van Damme P. EFNS guidelines on the clinical management of amyotrophic lateral sclerosis (MALS)–revised report of an EFNS task force. Eur J Neurol. (2012) 19:360–75. 10.1111/j.1468-1331.2011.03501.x 21914052

